# Clinical practice guidelines for diagnosis of autism spectrum disorder in adults and children in the UK: a narrative review

**DOI:** 10.1186/s12888-018-1800-1

**Published:** 2018-07-13

**Authors:** Jennie Hayes, Tamsin Ford, Hateem Rafeeque, Ginny Russell

**Affiliations:** University of Exeter Medical School, St Luke’s Campus, University of Exeter, Exeter, EX1 2LU UK

**Keywords:** Autism spectrum disorder, Diagnosis, Clinical guideline, Narrative review, Social factors, Diagnostic uncertainty, Clinical judgement

## Abstract

**Background:**

Research suggests that diagnostic procedures for Autism Spectrum Disorder are not consistent across practice and that diagnostic rates can be affected by contextual and social drivers. The purpose of this review was to consider how the content of clinical practice guidelines shapes diagnoses of Autism Spectrum Disorder in the UK; and investigate where, within those guidelines, social factors and influences are considered.

**Methods:**

We electronically searched multiple databases (NICE Evidence Base; TRIP; Social Policy and Practice; US National Guidelines Clearinghouse; HMIC; The Cochrane Library; Embase; Global health; Ovid; PsychARTICLES; PsychINFO) and relevant web sources (government, professional and regional NHS websites) for clinical practice guidelines. We extracted details of key diagnostic elements such as assessment process and diagnostic tools. A qualitative narrative analysis was conducted to identify social factors and influences.

**Results:**

Twenty-one documents were found and analysed. Guidelines varied in recommendations for use of diagnostic tools and assessment procedures. Although multidisciplinary assessment was identified as the ‘ideal’ assessment, some guidelines suggested in practice one experienced healthcare professional was sufficient. Social factors in operational, interactional and contextual areas added complexity to guidelines but there were few concrete recommendations as to how these factors should be operationalized for best diagnostic outcomes.

**Conclusion:**

Although individual guidelines appeared to present a coherent and systematic assessment process, they varied enough in their recommendations to make the choices available to healthcare professionals particularly complex and confusing. We recommend a more explicit acknowledgement of social factors in clinical practice guidelines with advice about how they should be managed and operationalised to enable more consistency of practice and transparency for those coming for diagnosis.

**Electronic supplementary material:**

The online version of this article (10.1186/s12888-018-1800-1) contains supplementary material, which is available to authorized users.

## Background

The diagnosis of autism poses particular challenges for healthcare professionals (HCPs) as, in common with other neurodevelopmental disorders and most psychiatric disorders, there are no biomarkers utilised in clinical practice [1–3]. In addition, the condition is heterogeneous, with wide ranging levels of severity and symptom expression and characteristics common to autism may occur in people with other conditions [4]. Those coming for diagnosis may also have symptoms of other conditions such as epilepsy, learning disability or sleep disorders, for example, complicating diagnosis further, with some arguing for a de-compartmentalisation of these conditions in younger children [5]. The ‘gold standard’ of diagnosis is considered to be consensus agreement within a multi-agency team [6, 7]. However, negotiating consensus between HCPs with different training, professional roles, experience and knowledge can be challenging and time consuming. Finally, a review of the accuracy, reliability, validity and utility of reported diagnostic tools and assessments found that many diagnostic instruments for autism lack a high-quality independent evidence base [6]. For example, only three instruments - the Autism Diagnostic Observation Schedule (ADOS), Autism Diagnostic Interview Revised (ADI-R) and the Childhood Autism Rating Scale (CARS) - had a strong supporting evidence base [6].

Given the potential challenges, clinical practice guidelines (CPGs) perform an important role in informing HCPs of best practice. CPGs are ‘systematically developed statements to assist practitioner and patient decisions about appropriate health care for specific clinical circumstances’ [8]. National CPGs in the UK help to provide evidence-based recommendations to support Autism Strategies and Action Plans [9] and form the guidance framework for HCPs undertaking assessment and diagnosis of autism in the UK. In addition to CPGs produced by specialist, government supported healthcare associations, for example, the Scottish Intercollegiate Guidelines Network (SIGN) [10], professional clinical bodies also publish discipline-specific practice parameters or position papers, for example, the Royal College of Psychiatrists (RCPsych) [11].

### Social factors

Although CPGs aim to inform diagnostic practice, research suggests that diagnostic and assessment procedures vary in practice [9]. Diagnosis is dependent on observing socially-based behaviours that are arguably not necessarily characteristic of the person under assessment but arise from two-way social relationships and social context. Assessment mechanisms include drawing information from a range of sources, including clinician observation, reporting from family members and wider contexts such as school or workplace. This means that assessments are contextual and inter-relational and symptoms may change according to context or interpersonal relationship, making different assessment sources potentially contradictory.

Some studies show that social factors such as individual patient preference, availability of resources or local organisational factors can shape diagnostic practice, in, for example, heart disease [12]. Studies in autism have also shown how diagnostic rates can be affected by contextual and social drivers, such as diagnostic resources [13] or diffusion of information about autism through social networks [14]. Where there is diagnostic uncertainty clinicians may ‘upgrade’ to a diagnosis of autism if they believe it would be in the best interests of the patient; if the diagnosis would trigger appropriate services and funding; or counteract the limitations of diagnostic tools, particularly in atypical presentations [15, 16]. It seems, in practice, clinicians may adopt a pragmatic, practical or functional approach.

### Socio-economic and cultural factors

Research has shown that lower social economic status (SES) is associated with increased parent-reported prevalence [17], contrasting with the US where higher SES and parental education is linked to increased likelihood of diagnosis [14, 18]. Research also suggests that people with autism from Black, Asian and Minority Ethnic (BAME) communities are less likely to be diagnosed with autism or access appropriate services [19] despite research which shows that behaviours associated with autism are likely to be consistent across cultures and countries [20].

Prior to diagnosis, social factors can also determine who comes forward for diagnosis and who is referred for further assessment. Research examining a longitudinal UK cohort study identified that with the severity of autistic traits held constant, younger mothers and mothers of first-born children were significantly less likely to have children diagnosed with ASD [21]. In addition, boys were more likely to receive a diagnosis than girls, and maternal depression was linked with a lack of diagnosis [21]. These findings suggest both cultural and economic influences impact the diagnostic pathway.

### Biomarkers in autism diagnosis

There is a great deal of research that explores the underlying neurobiological, genetic, chemical and cognitive factors that may, in future, provide biomarkers which could be utilised in autism diagnosis (see [22] for a review of genetic, metabolic and brain focused biomarkers). For example, a recent research study has identified a link between damage to proteins in blood plasma and autism symptoms [23]; while another found shared brain activity between boys diagnosed with ASD and those with obsessive-compulsive disorder (OCD) which in turn differed from a non-diagnosed control group [24]. However, it has been argued that the heterogeneous and interactive nature of autism symptoms makes the identification of clinically useful biomarker tests problematic [25]. Furthermore, findings from biomarker research have yet to be integrated with clinical practice and none currently have enough evidence to support routine clinical use [22]. For the foreseeable future, therefore, these developments are unlikely to change diagnostic practice [26].

### Purpose of the review

Although a few studies have begun to explore health professionals’ views of autism diagnosis [16, 27, 28], to our knowledge there are few studies that examine how clinical guidelines may inform assessment. One exception is a recent systematic review of English speaking guidelines undertaken by Penner et al. [29] which reported that guidelines varied considerably in quality, content and recommendations but included guidelines working across incomparable health systems in different countries. We therefore carried out a focussed narrative review of guidelines that impact on UK-based practice. Penner et al. suggest that in the face of disparate clinical guidance clinicians should ‘be mindful of local resources and wait times, eligibility requirements for ASD services…and the wishes of families when deciding on how best to assess for ASD’ [29]. Our narrative review responds to this call for a pragmatic approach by investigating where, within guidelines, social factors and influences such as those suggested are considered.

## Method

### Scoping search

A scoping search was undertaken to check there was no similar review published. A search was made in the following databases; PsychARTICLES; Embase; Global health; HMIC; Ovid (books; medline; journals); PsychINFO; Social policy and practice. One relevant article was retrieved [29], as discussed above.

### Inclusion and exclusion criteria

Full inclusion and exclusion criteria are in Table 1. Whilst we took a broad approach to CPGs, including, for example, journal articles summarizing national CPGs and the diagnostic process, as well as national CPGs, the researchers acknowledge that each of these type of guidelines have different purposes (see Table 2). However, we argue that each may have an impact on HCP’s process of diagnosis to a greater or lesser extent and for the purposes of this study all were included under the term clinical practice guidelines.

**Table 1:** Inclusion and Exclusion Criteria

Inclusion Criteria	
Documents with guidance-based status for HCPs working in secondary care in the UK; or were published papers, aimed at HCPs, with the aim of reviewing CPGs	
Documents related to autism diagnosis and assessment for either children, adults or both	
Documents produced either by or through government or professional clinical bodies or published in a journal aimed at HCPs	
Documents related to diagnosis and assessment in UK (England, Scotland, Wales and N Ireland)	
Documents dated from 2009 (reflecting publication of the first UK specific Autism Act) or were the most recent CPG published by a key professional body	
Exclusion Criteria	
Documents related solely to referral, treatment, prognosis or support services	
Reviews of diagnostic criteria and other academic papers	
Guidelines related to primary care as we were interested in diagnosis rather than referral	
Narrative reviews, editorials and opinions	
Documents related to parliament or legislature; national or regional strategies as they are not the primary source for clinicians	
Local guidance	
Guidance provided by private providers of diagnostic services	
International professional body guidelines (other than ICD/DSM)

**Table 2:** Purpose of Diagnostic Guidelines

Type of guideline	General purpose of type of guideline
Diagnostic Criteria	To assist clinicians in the diagnosis of mental conditions by providing descriptions of the main clinical features in each category
National Clinical Guidelines	To offer best practice advice and guidance for professionals and service users and their families
Guidelines from Professional Bodies	To offer profession specific advice to clinicians and healthcare professionals in their specialist area
Journal Articles	To summarise clinical guidelines in clinician-facing publications to keep clinicians up to date and/or alert them to changes in good practice

### Identification of CPGs

**Fig. 1:**
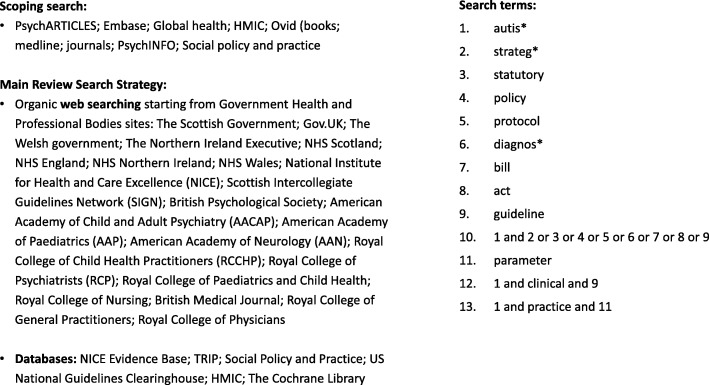
Full Search Strategy

We did not set out to undertake a comprehensive systematic review, as it was not a requirement of our study that we consider risk of bias either within or across studies [30]. However, we took a PRISMA approach to our search strategy, borrowing from systematic review methodology in terms of screening titles and abstracts and data extraction techniques [31]. A systematic search was conducted in June 2017 using the following databases: NICE Evidence Base; TRIP; Social Policy and Practice; US National Guidelines Clearinghouse; HMIC; The Cochrane Library. In addition, searches were made of government related websites and relevant professional bodies as well as NICE and SIGN. We used the following search terms to search all databases and websites: ‘autism’, ‘diagnosis’, ‘guidance’, ‘statutory’, ‘clinical’, ‘practice’, ‘guideline’, ‘protocol’, ‘strategy’, ‘policy’, ‘bill’, ‘act’, and ‘parameter’. A full search strategy is in Fig. 1.

### Study selection

**Fig. 2:**
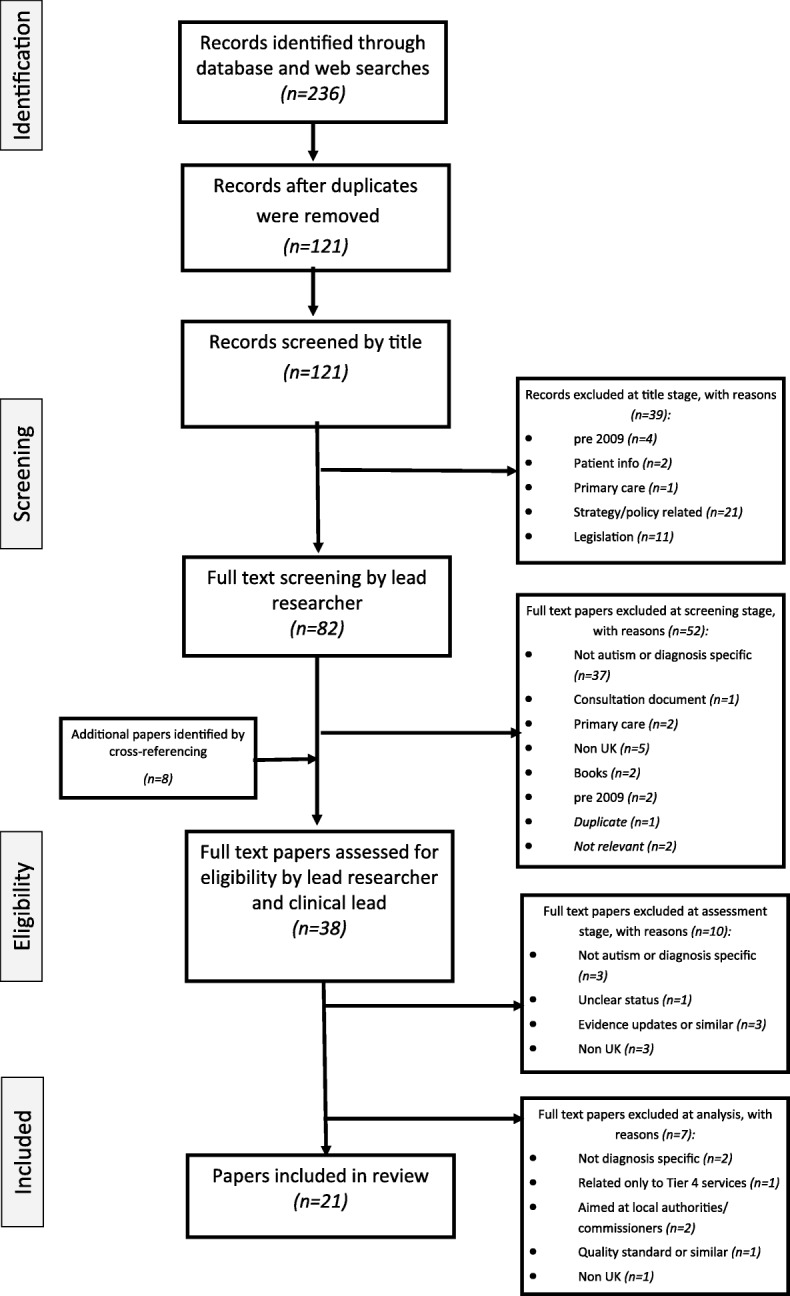
Study selection flow diagram

The first reviewer (JH) removed duplicates and screened titles for relevance. Full text copies of the potentially relevant documents were downloaded for screening. The first reviewer screened full text documents and excluded those not relevant. The remaining titles were independently checked by the clinical specialist (TF) using pre-specified inclusion/exclusion criteria (outlined in Table 1). Discrepancies were resolved by discussion, with involvement of a third reviewer (GR). Twenty-eight documents were considered for analysis, with seven being withdrawn at full analysis stage. See Fig. 2 for full details.

Guidelines from the International Classification of Mental and Behavioural Disorders (Tenth edition) (ICD-10) [32] and the Diagnostic and Statistical Manual of Mental Disorders (Fifth edition) (DSM-5) [33] were considered alongside UK relevant guidelines as they are considered authoritative sources for the definition of symptoms utilized in autism diagnosis, as well as other neurological conditions.

### Data extraction

A data extraction framework was created to draw key characteristics from the guidelines (year, author, geographical remit, target audience, age range, range of diagnoses covered, age at which symptoms are recognised, diagnostic criteria referred to); as well as key elements in the diagnostic process (recommended tools, role and composition of the multidisciplinary team (MDT), who can diagnose, assessment targets and key features of assessment). This framework was piloted with four reviewers (JH, GR, RW and DE) in a comparison of analysis of three guidelines. The framework was amended accordingly and is included in Additional file 1. Data were independently extracted by two reviewers (JH and HR) from 21 CPGs and disagreements were resolved by discussion and further checks. Data were tabulated and analysed.

### Analysis of social factors

A modified form of narrative review, as described by Popay et al. [34] and Ferrari [35], was adopted whereby data extraction enabled synthesis of key data, whilst also allowing rich narrative description [35]. Narrative review was selected as it enabled the telling of the ‘story’ of CPGs, and consideration of how guidelines, as a set of texts, shape diagnosis [34].

A process of inductive analysis was undertaken based on social factors and influences. These were defined, for the purpose of this review, as contextual factors that influence diagnosis but are not based on symptoms of autism. We drew from the concept of a social model of diagnosis as developed by Jutel and Nettleton [36]. This model considers how diagnostic classifications and medical diagnoses are socially created and how social forces – including technological, professional, cultural and economic forces – contribute to shaping aspects of the diagnostic process including those related to classification, the consequences of diagnosis and the process of diagnosis itself [36]. Overall, a social model challenges the idea of diagnosis as ‘a moment of clinical purity’ [37] or as a way simply to identify underlying biological problems. We included factors that were relevant to multidisciplinary working or parental/family influence (the process of diagnosis); the potential outcomes of diagnosis for the patient and how HCPs may take this into account (the consequences of diagnosis); and how issues around classification shape the diagnostic process such as how borderline cases are dealt with (diagnosis as a category). This was a dynamic process whereby data extracts were considered in relation to each other via conceptual mapping and clustering [34].

### Terminology

For the purposes of this review and in line with the Autism Strategy [38] we use the term ‘autism’ throughout.

## Results

### Characteristics of guidelines

A total of 236 documents were retrieved, and 21 were included in the final narrative review (see Table 3 for full list of included documents and guideline characteristics). The documents studied are grouped into four types: a) International Diagnostic Criteria (*n* = 2); b) National Clinical Guidelines (*n* = 5); c) Journal articles that summarize National Clinical Guidelines and the diagnostic process, published in key clinical journals (*n* = 10); d) Guidelines from professional bodies (*n* = 4). It should be noted that journal articles, in some cases, are designed to give an update rather than a full guideline therefore the lack of detail in some areas should not necessarily be seen as a weakness.

**Table 3:** Key characteristics of guidelines

Title	Year	Author(s)	Publisher/Journal	Geographical remit	Target audience	Age range	Range of diagnoses covered	Diagnostic criteria referred to	Age at which symptoms are recognised
**DIAGNOSTIC CRITERIA**									
The ICD-10 Classification of Mental and Behavioural Disorders: clinical descriptions and diagnostic guidelines [32]	1993	N/A	World Health Organisation	International	Clinical, educational and service use	All ages	Pervasive development disorders	N/A	Before age of 3 years (childhood autism); after age 3 (atypical autism).
Diagnostic and Statistical Manual of Mental Disorders (Fifth Edition) [33]	2013	N/A	American Psychiatric Association	International	Clinicians, students, practitioners, researchers	All ages	Autism Spectrum Disorder	N/A	During 2nd year of life (12–24 months) or earlier than 12 months if developmental delays are severe
**NATIONAL CLINICAL GUIDELINES**									
NICE Autism in under 19 s: recognition, referral and diagnosis (NICE CG128) [39]	2011	National Collaborating Centre for Women’s and Children’s Health	National Institute for Health and Care Excellence (NICE)	England and Wales	Healthcare professionals	From birth up to 19 years	Pervasive developmental disorder (PDD)	ICD-10 or DSM-IV	May be uncertainty before 24 months, or with developmental age of less than 18 months
Six Steps of Autism Care for children and young people in Northern Ireland (RASDN) [44]	2011	Regional Autistic Disorder Network for Northern Ireland	Health and Social Care Board	Northern Ireland	Health care and education professionals, parents, carers, service users and providers.	Up to the age of 18 years	Autism spectrum disorder	ICD-10, DSM-IV, NICE, SIGN, NZ Guidelines, NHS Map of Medicine	Pre-school. Language delay by the age of two years.
Autism Spectrum Disorder in adults: diagnosis and management (NICE CG142) [9]	2012	National Collaborating Centre for Mental Health	National Institute for Health and Care Excellence (NICE)	England and Wales	Health and social care providers and commissioners	Adults aged 18 and over	Autism spectrum disorders	N/S *ICD-10 specified in full version of CG142 [62]	N/A
Autism Adult Care Pathway (RASDN) [54]	2013	Regional Autistic Spectrum Disorder Network	Health and Social Care Board	Northern Ireland	Professionals, adults and families	Adults from age 18	Autism spectrum disorders	DSM-5 and ICD-10, NICE guidance CG142.	N/S
Assessment, diagnosis and interventions for autism spectrum disorders: A national clinical guideline (SIGN 145) [10]	2016	N/A	Scottish Intercollegiate Guidelines Network	Scotland	Healthcare professionals	Whole age range	Autism spectrum disorder	ICD-10 and DSM-5	Autism can be reliably diagnosed between the ages of 2–3.
**GUIDELINES FROM PROFESSIONAL BODIES**									
RCSLT (Royal College of Speech and Language Therapists Clinical Guidelines (Autism) [41]^a^	2005	N/A	Royal College of Speech and Language Therapists	UK	Speech and language therapists	Children and adults	Autism spectrum disorder	ICIDH-2 *(for general clinical assessment)*	N/S
Good practice in the management of autism (including Asperger syndrome) in adults (RCPych CR191) [11]	2014	Royal College of Psychiatrists	Royal College of Psychiatrists	UK	Psychiatrists working with adults of at least normal intellectual ability	Adults from age 18	Autism	ICD-10, DSM-5, NICE, 2012.	N/S
Autism Spectrum Disorders: Guidance for Psychologists (BPS) [40]^b^	2016	Stuart-Hamilton, Dillenburger, Hood & Austin	British Psychological Society	UK	Psychologists	All ages	Autism Spectrum Disorder	ICD-10 and DSM-5, NICE, 2011.	Both diagnostic manuals consider ASD indicators to be present by the age of 36 months although some children can be identified under the age of 24 months.
BMJ Best Practice online resource [43]	2017	Parr &Woodbury-Smith	British Medical Journal	Outside US and Canada	Medical Practitioners	All ages	Autism Spectrum Disorder	DSM-IV, DSM-5 & ICD-10. NICE, SIGN, AACAP, AAP, NZ ASD guideline, AAN	More than 80% of children with ASD show clear behavioural signs by the age of 24 months, some indicators in 12–18 months
**JOURNAL ARTICLES**									
Diagnosis and management of autism in childhood [47]	2011	Blenner, Reddy & Augustyn	British Medical Journal	N/S	General clinicians	Children	Autism Spectrum Disorder	DSM-IV TR or ICD-10	N/S
Diagnosis and assessment in autism spectrum disorders [48]	2012	Carpenter	Advances in Mental Health and Intellectual disabilities	N/S	Those designing and providing diagnostic services	All ages	Autism Spectrum Disorder	DSM-IV TR or ICD-10. Gillberg’s for AS. There are others but few use them (Kopra et al., 2008; Chiappedi et al., 2010).	N/S
Autism spectrum disorder in adults: clinical features and the role of the psychiatrist [49]	2013	Garland, O’Rourke & Robertson	Advances in Psychiatric Treatment	UK	Psychiatrists	Adults	Autism Spectrum Disorders	ICD-10 and DSM-5, NICE	To satisfy ICD-10 criteria for childhood autism, impairments must manifest before the age of 3 years
Recognising, referring and diagnosing autism [45]	2012	Howlett & Richman	Every Child Journal	England and Wales	Professionals working with children and young people	Children and young people	Autism	NICE	The core autism behaviours are typically present in early childhood; but features can appear different with age or change with circumstances
Autism [50]	2013	Lai, Lombardo & Baron-Cohen	The Lancet	N/S	N/S	All ages	Autism or the autism spectrum	DSM-5, ICD-10	N/S
Autism [51]	2009	Levy, Mandell & Schultz	The Lancet	N/S	N/S	N/S but primarily talks about children	Autism Spectrum Disorder	DSM-IV and ICD-10	Parents often aware from age 18 months, a diagnosis is often not made until 2 years after the initial expression of parental concern.
Autism spectrum disorder: diagnosis and management [53]	2009	O’Hare	Archives of Disease in Childhood: Education and Practice Edition	N/S but relates primarily to SIGN guidelines	Paediatricians	Children and young people	Autism Spectrum Disorder	ICD-10 and DSM-IV, SIGN	N/S
Recognition, referral, diagnosis, and management of adults with autism: summary of NICE guidance [58]	2012	Pilling, Baron-Cohen, Megnin-Viggars, Lee & Taylor	British Medical Journal	England and Wales	N/S	Adults	Autism	N/S	N/S
Autism Spectrum Disorders in childhood: a clinical update [46]	2011	Reynolds	Community Practitioner	UK	Community practitioners	Children	Autism Spectrum Disorder	ICD-10, DSM-IV	N/S
The NICE guideline on recognition, referral, diagnosis and management of adults on the autism spectrum [52]	2014	Wilson, Roberts, Gillan, Ohlsen, Robertson & Zinkstok	Advances in Mental Health and Intellectual Disabilities	England and Wales	Health care professionals, service managers, service users, practitioners	All adults	Autism spectrum disorder	N/S	N/S

^a^Pre 2009 but constitutes current guideline in use from RCSLT

^b^Currently under review but represents the most recent published guideline from BPS

Of the 21 guidelines considered, six dealt with diagnosis of adults, seven with children and eight with all ages. Of those, two guidelines were international but key to diagnostic practice in the UK (ICD-10 and DSM-5), five related to the UK as a whole, five to England and Wales, one to Scotland, two to Northern Ireland and one to outside the US and Canada (and therefore included the UK). Five guidelines did not specify a geographical remit but were published in the UK in clinician-facing journals. All guidelines were aimed at HCPs, with six aimed at particular specialist roles that included psychiatrists, psychologists, speech and language therapists, community practitioners and paediatricians.

Guidelines acknowledged that there is variation in rates of identification, assessment criteria and practice [9]; that there is increasing demand for diagnostic services [39]; and that increased awareness of autism is likely to lead to a rise in people presenting for assessment [40].

### Definitions of autism

Definitions of autism in ICD-10 and DSM-5 differed. ICD-10 took a categorical approach with a definition of Pervasive Development Disorders that included sub-diagnoses within it; whilst DSM-5 used the overarching dimensional concept of Autism Spectrum Disorder. Some inconsistencies were present related to the differences in classification in ICD-10 and DSM-5, therefore, for example, Rett’s Syndrome and Asperger’s Syndrome were sub-diagnoses of Pervasive Development Disorders in ICD-10, but were encompassed in the overarching diagnosis of Autism Spectrum Disorder in DSM-5 [32, 33]. Definitions of autism in all other guidelines considered in this study were broadly consistent with the idea of a ‘spectrum’.

Most guidelines (*n* = 14) referred to symptom criteria from both ICD-10 and the (then) current version of DSM (DSM-IV up to 2012 and DSM-5 from 2013), with eight guidelines recommending that HCPs should use the current version of DSM or ICD criteria for diagnosis. Exceptions were NICE CG142, which was based on ICD-10, [9]; Royal College of Speech and Language Therapists (RCSLT) [41], which drew on the International Classification of Functioning, Disability and Health (ICIDH-2) for general clinical assessment [42]; and journal articles describing NICE guidelines which made no mention of DSM/ICD (*n* = 3).

Overall, therefore, the guidelines were mixed in their recommended sources for symptom criteria due to the current differences in the two classification systems.

### Narrative review of social factors

**Fig. 3:**
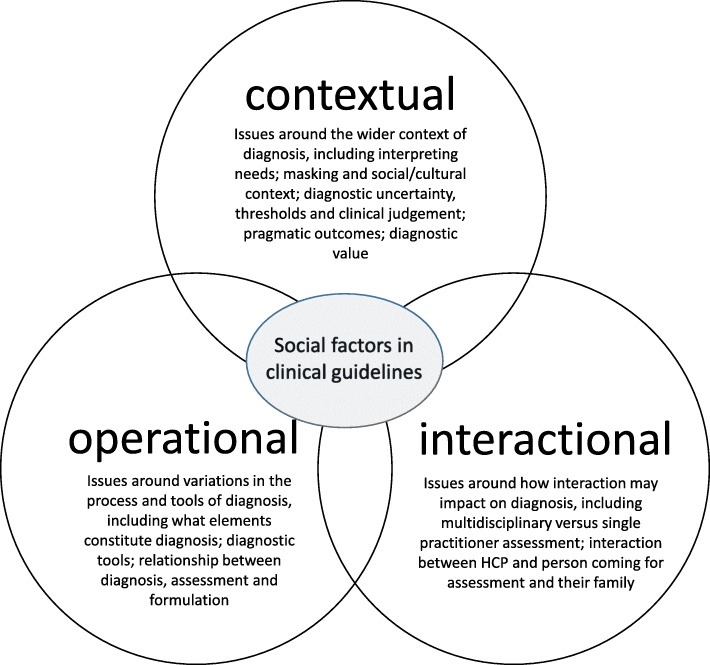
Social factors in clinical guidelines

We used three inter-related elements as an organising framework to describe the social factors identified in clinical guidelines: operational, interactional and contextual. These factors do not stand alone from each other, indeed, they appear to have a dynamic and inter-dependent relationship, however, organising them provides a way to map their range and scope (see Fig. 3).

### Operational factors

Operational factors included how different assessment processes impact on the diagnostic decision, such as which tools and processes are engaged and when; what constitutes an assessment; and whether the decisions take place as part of diagnosis or formulation. Table 4 outlines some of these operational factors.

**Table 4:** Key diagnostic recommendations

CPG	Recommended tools	MDT recommended	MDT membership	Assessment targets	Key features of assessment
**DIAGNOSTIC CRITERIA**					
ICD-10 (1993) [32]	N/S	N/S	N/S	N/S	Diagnose on the basis of behavioural features
DSM-5 (2013) [33]	No specific tool	N/S	N/S	N/S	Careful clinical history & summary of social, psychological & biological factors. Multiple sources of information: • clinician’s observations • caregiver history • self-report (where possible) Clinical judgement
**NATIONAL CLINICAL GUIDELINES**					
NICE CG128 (2011) [39]	No specific tool recommended	Autism team members should carry out assessment (short version). A diagnosis can be made by a single experienced HCP; profile of strengths & weaknesses is essential, and requires MDT [55] (full version).	Autism team made up of Paediatrician &/or Child & Adolescent Psychiatrist, SLT, Clinical &/or Educational Psychologist & access to paediatrician/paediatric neurologist, Child & Adolescent Psychiatrist, Educational Psychologist, Clinical Psychologist, OT, if not in team. Also consider specialist health visitor or nurse, specialist teacher or social worker.	Start the autism diagnostic assessment within 3 months of referral. Follow up appointment within 6 weeks of assessment.	Seek report from the pre-school or school; gather additional health or social care information. Include in every autism diagnostic assessment: • questions about parent/carer/child’s concerns • details of the child’s experiences of home life, education and social care • developmental history, focusing on developmental and behavioural features • assessment (through interaction with and observation of the child or young person) of social and communication skills and behaviours • medical history, including prenatal, perinatal and family history, and past and current health conditions • physical examination • consideration of the differential diagnosis • systematic assessment for conditions that may coexist with autism • development of a profile of the child’s or young person’s strengths, skills, impairments and needs that can be used to create a needs-based management plan, taking into account family and educational context • communication of assessment findings to the parent/carer/child
RASDN (2011) [44]	No specific tool	The use of MDT approach is necessary	Involving at least two disciplines: paediatrician; child psychiatrist; SLT, OT, clinical psychologist; specialist health visitor; mental health practitioner (CAMHS); social worker; nurse; ed. psych. Teacher; other trained professionals	Referral screened within 5 days. Info provided within 4 weeks. 13 weeks to first appointment. Feedback within 4 weeks, report within 6 weeks of formulation.	Step one: Initial directed conversation. Step two: Integrated multidisciplinary team assessment (leads to diagnosis/non-diagnosis) includes: • medical history inc: birth history, family history, & general medical concerns • developmental history focusing on developmental & behavioural concerns • observational assessment of the child/young person • further assessment/observations in another setting (school/home) • physical exam in some groups • specific assessments may be required, e.g. SLT assessment • educational assessment Step three: Integrated MDT formulation (leads to wider understanding of difficulties) Step four: family feedback and care planning
NICE CG142 (2012) [9]	Identification: Consider AQ-10 (without LD); Brief assessment (with LD). Diagnosis and assessment: AAA including AQ and EQ; ADI-R; ADOS-G; ASDI; RAADS-R (without LD). ADOS-G; ADI-R (with LD); DISCO, ADOS-G, ADI-R	Comprehensive assessment should be team based (short version). At a minimum by a qualified clinician usually a clinical psychologist, psychiatrist or neurologist [62] (full version).	Specialist autism team made up of: Clinical Psychologists, Nurses, OTs, Psychiatrists, Social Workers, SLTs, Support Staff	N/S	During a comprehensive assessment, enquire about and assess the following: • core autism signs and symptoms that have been present in childhood and continuing into adulthood • early developmental history, where possible • behavioural problems • functioning at home, in education or in employment • past and current physical and mental disorders • other neurodevelopmental conditions • hyper- and/or hypo-sensory sensitivities and attention to detail. Direct observation of core autism signs and symptoms especially in social situations. Assess for possible differential diagnoses and coexisting disorders Assess risks; Develop care plan, provide health passport, consider 24 h crisis management plan; Assess challenging behaviour Consider further investigations on individual basis
RASDN (2013) [54]	Screening: GADS, GARS-2, AASQ, ASAS, NAS, AQ-10 History: ADI-R, DISCO, ASDI, RAADS-R; Direct assessment: ASIT, HSST, SSQ, Observation: ADOS-G	Diagnosis must be team based & draw on a range of professionals.	At least two of: clinical psychology (core), psychiatry, SLT, LD/MH nursing; OT, other appropriately trained professionals.	Final report to be provided within 6 weeks of assessment.	As an absolute minimum, elements 2, 3 & 4 *must* be included in the assessment. 1. Neurodevelopmental history, corroborated via relative/family; 2. Direct autism specific assessment with individual; 3. Observational recording of assessment sessions; 4. Clinical judgement. May also include; standardized measure of adaptive functioning; assessment of language & communication skills; functional assessment of problematic behaviour; full needs assessment
SIGN 145 (2016) [10]	Identification: AQ-10 Diagnosis and Assessment: E.g. ADI-R, DISCO, 3di, CARS, CARS-2, ADOS-G. NAPC and RCPsych guides.	MDT … should be considered as the optimum approach	Experienced professionals	N/S	• History taking (informant interview): prenatal, perinatal & developmental history; description of the current problems experienced; family history; description of who is in family; coexisting conditions and differential diagnoses • Clinical observation/assessment (individual assessment/interview): directly observe & assess the individual’s social & communication skills and behaviour • Contextual and functional information from a variety of settings and people • Profile of the individual’s strengths and difficulties: communication, cognitive, neuropsychological and adaptive functioning; motor and sensory skills • Biomedical investigations on an individual basis when clinically relevant • Assessment of mental health needs, wellbeing and risk should be considered
**GUIDELINES FROM PROFESSIONAL BODIES**					
RCSLT (2005) [41]	N/S	Should always be multidisciplinary & multi-agency to achieve optimum benefit.	This may include SLT, child psychology, child psychiatry, clinical psychology, paediatrician, EdPsych., OT & teacher	N/S	During assessment, consideration must be given to the triad of social impairments, as well as theories relating to the triad, for example sensory sensitivity and integration; intersubjectivity; executive functioning deficits; motivation; memory and central coherence. • Joint attention • Readiness & ability to focus & shift attention • Social interaction • Use of communicative strategies • Evaluation of child’s play • Info about learning potential • Impact of individual’s mental health
RCPsych (2014) [11]	Identification: AQ, RAADS-R. RPsych Guide. Questionnaires: ASAS, GARS, GARS-2, SCQ, SRS-2, AQ, AQ-10, RAADS-R, SCDS, ABC. Diagnostic interviews: ADI-R, ADOS-2, DISCO, 3Di, AAA, RPsych Guide, PDD-MRS, ASDI, CARS-2, HBS, WADIC Assessment for associated dev disabilities: AQ, EQ, SQ, Faces test, eyes test, Faux Pas Recognition Test, SSQ, Dewey’s Social Stories, Adult/Adolescent sensory profile	NICE advocates multidisciplinary exercise, but psychiatrists might be expected to diagnose straightforward cases & be alert to indications for a more specialist assessment.	MDT usually includes psychology & nursing as core membership	N/S	• Speak with informant • Take neurodevelopmental history • Consider obtaining early health records Might include assessment for; cognitive ability, functional ability, coexistent neurodevelopmental disabilities, coexistent psychiatric disorders, mental capacity, risk of harm/offending, medical problems Wherever possible, it is essential that the clinician gets accurate accounts of relationships in different settings (e.g. at work & at home), particularly where they might be more demanding for that individual.
BPS (2016) [40]	e.g. ADOS, ADI, DISCO, ADI-R	It is recommended that assessment is multidisciplinary.	At least one psychologist, in addition to other relevant personnel, such as OTs, mental health workers etc.	It is recommended that assessment is timely.	The taking of a developmental history with carers as well as observation across different settings. Information from a range of sources. Psychologists contribution to identification and assessment may include: • Assessment of protective factors, strengths and abilities • Assessment of associated mental health issues • Comprehensive developmental and family history • Assessment of learning styles • Assessment of strengths and of barriers to learning • Assessment of environmental conditions for learning • Functional behavioural assessment • Assessment of social communication style • Assessment of the needs of families. • Comprehensive cognitive assessment, which may include psychometrics if deemed necessary
BMJ (2017) [43]	Screening: CHAT, M-CHAT Parental questionnaires: SCQ, CAST, CARS; for adults, the SRS, ASQ. Diagnosis and Assessment: eg ADOS-G, ADI-R; 3di; DISCO	Diagnosis should be confirmed or made by an appropriately trained professional, ideally working as part of MDT	Paediatricians, child psychiatrists, adult psychiatrists or psychologists, & other professionals	N/S	A combination of: • neurodevelopmental history • standardised interview, & • observational assessment Gather information about functioning in more than one environment; A full neurological examination including measurement of head circumference is routinely performed in all children.
**JOURNAL ARTICLES**					
Blenner et al (2011) [47]	Screening: CHAT, PDDST, STAT, CHAT-23, M-CHAT, ITC, SCQ. Diagnosis: ADOS.	Paediatric neurologists, developmental & behavioural paediatricians, child psychiatrists or psychologists, or, ideally, MDT.	N/S	N/S	Comprehensive evaluation that includes • lifetime & family history • review of medical & educational records • behavioural observation • physical examination • administration of standardised instruments such as the autism diagnostic observation schedule • cognitive & adaptive assessment • review of established DSM or ICD diagnostic criteria • Assessment of specific domains, such as communication skills, sensory and motor problems, and family stressors and coping abilities • Look for causes & co-occuring conditions (further tests)
Carpenter (2012) [48]	Screening: ASDASQ, AQ and EQ, AAA. AQ-10, RAADS-R. RCPsych guide. Observation: PDD-MRS (with ID); ADOS-G. Interview: ADI-R, DISCO, 3Di. AAA to provide structure.	Diagnosis can be made by one clinician. Wider assessment requires a team. A variety of professionals can diagnose.	N/S	Labour intensive - up to 8 h to make & document diagnosis.	Three elements (judged against criteria of ICD-10 or DSM-4): • interview with person • observation • interview with an informant Some clinicians bypass the criteria & test, for example, theory of mind, central coherence. Consider possible co-morbidities Holistic assessments needs to be structured around: • Need for social support and for help with employment • Sensory and processing difficulties • Medical issues • Neuro-psychiatric conditions • Practical skills, including motor difficulties • Social interaction skills • Emotional understanding (of self and others) and personal coping strategies • Interests and preoccupations • Sexual interests and future desires • Insight and future desires and motivation • Psychiatric concerns • Other behaviours that may get person into contact with the law • Support for carers
Garland et al. (2013) [49]	Screening: AQ-50, AQ-10 Diagnosis: ADI-R, ADOS = G, RCPsych Diagnostic Interview Guide	When mental health difficulties also exist, the expertise of the wider MDT is likely to be engaged.	Outlines psychiatrist’s role.	Enough time should be set aside	• History of presenting complaint • Psychiatric history • Family history • Medical history • Developmental history • Personal & social history • Mental state examination • Assess for comorbid disorders inc. neurodevelopment disorders • Physical assessment • Functional level assessment • Assess risk • Assessment of care & support needs • Consideration of need in areas of education & employment
Howlett & Richman (2011) [45]	No specific tool	If the local autism team does not have the skills to assess these children themselves, they should liaise with professionals who are able to do so	Minimum, paediatrician &/or child & adolescent psychiatrist, SLT & clinical &/or Ed.Psych. Other professionals … specialist health visitor, nurse, specialist teacher, social worker	Timely & appropriate. Follow up appointment within six weeks of assessment	Should provide detailed developmental profile. Based on NICE guidance.
Lai et al....... (2013) [50]	Screening: CHAT, ESAT, M-CHAT, ITC, Q-CHAT, STAT (for young children); SCQ, SRS, SRS-2, CAST, ASSQ, AQ (for older children and adolescents); AQ, RAADS-R (FOR ADULTS). Diagnosis and assessment: ADI-R, DISCO, 3Di (for structured interview); ADOS, ADOS-2, CARS, CARS-2 (observational measure).	Assessment needs to be multidisciplinary	N/S	N/S	• Interview with the parent or caregiver • Interaction with the individual • Collection of information about behaviour in community settings • Cognitive assessments • Medical examination • Co-occurring conditions
Levy et al (2009) [51]	SCREENING: Q-CHAT, M-CHAT, FYI, ECI-4, CSI-4, SCQ, ASDS, KADI, AQ-Child, A (AUTISM) ABC (autism), PDDRS, PDD-MRS, DBC, DBC-ES, PDDBI, ABC (aberrant), CCC, SRS, RBS-R, SCDC. Diagnosis and assessment: PIA-CV, DISCO, ADI-R, 3Di. CHAT, STAT, AOSI, ADOS, CARS	These assessments should be multidisciplinary	The MDT should include clinicians skilled in speech & language therapy, occupational therapy, education, psychology, & social work.		• Use ICD or DSM criteria • Core and comorbid symptoms, cognition, language, & adaptive, sensory, & motor skills. • Review of caregiver concerns, descriptions of behaviour, medical history, & questionnaires. • Include stage 1 data. • Observations across settings • Cognitive, communication, & ASD-specific assessment • Medical assessment • Differential diagnosis
O’Hare (2009) [53]	Screening: M-CHAT, NAPC Checklist Diagnosis: ADOS-G, SRS	A multidisciplinary diagnostic approach is recommended	Paediatricians are essential members.	N/S	• Direct clinical structured observations • Critical that information is gathered from different settings, outwith the clinic – there are structured questionnaires for parents/teachers • Physical exam and other specialist tests as required
Pilling et al. (2012) [58]	Identification: AQ-10.	N/S	N/S	N/S	Inquire about & assess the following: • Core autism signs & symptoms • Early developmental history • Behavioural problems • Functioning at home, education, employment • Past & current physical & mental disorders • Other neurodevelopmental conditions • Neurological disorders (for example, epilepsy) • Communication difficulties • Hypersensory &/or hyposensory sensitivities & attention to detail • Carry out direct observation of core autism signs & symptoms especially in social situations • Functional analysis
Reynolds (2011) [46]	No specific tool	N/S	N/S	N/S	Observed behaviours with patient presenting symptoms from ‘Triad of Impairments’: social interaction, social communication, social imagination
Wilson et al (2013) [52]	Identification: AQ-10 Diagnosis and assessment: ADI-R; ADOS-G. AAA, ADI-R, ADOS-G, ASDI, RAADS-R (without ID). ADI-R and ADOS-G (with ID). DISCO, ADI-R, or ADOS-G.	Should be carried out by MDT consisting of professionals who have experience in diagnosing autism (from NICE).	N/S	N/S	A comprehensive assessment of autism should involve an assessment of • core autism signs and symptoms • early developmental history, where possible, and in the absence of an informant written information, such as school reports may be used • behavioural problems • functioning at home, in education, or in employment • past and current physical and mental disorders • other neurodevelopmental conditions • neurological disorders (e.g. epilepsy) • sensory processing and sensory sensitivity issues Assess coexisting mental health disorders. Risk assessment. Functional analysis for challenging behaviour

Key OT Occupational Therapist, *SLT* Speech and Language Therapist, *HCP* Healthcare professional, *MDT* Multidisciplinary team, Ed.Psych Educational Psychologist

#### The assessment process

One guideline suggested that clinical practice varies greatly [43] and we found this to be mirrored in CPGs with a wide range of potential assessment processes included. DSM-5 recommended that a diagnostic assessment should include gathering multiple sources of information from clinician’s observations, caregiver history and self-report (where possible). National guidelines, although providing far greater detail, tended to include these areas and additionally suggested various other detailed assessments such as gathering wider functional/assessment information [10]; using documentary evidence, assessing risks, and assessment of challenging behaviour [9]; assessing for co-conditions [9, 39]; physical examination [39]; comprehensive educational assessment [44]; assessment of communication, neuropsychological functioning, motor and sensory skills, and adaptive functioning [10]. Professional guidelines added other factors such as comprehensive cognitive assessment [40] and impact of individual’s mental health [41], accounts of relationships in different settings [11] and observation in school or another setting [43]. Journal articles tended to reflect national guidelines and varied in the level of detail outlined for assessment factors. Two articles gave little detail of assessment processes but one referred readers directly to NICE guidelines for further detail [45] and the other was aimed at community practitioners who would be more likely to be involved in referral than diagnosis [46]. Articles also included assessment of co-occuring conditions (e.g. [47–52]) and a physical or medical examination (e.g. [47, 50]). Additional assessment areas included assessment of specific domains such as family stressors and coping abilities [47]. In one guideline [48] it was suggested that some clinicians bypass ICD/DSM criteria and instead undertake:

‘…testing for specific underlying difficulties such as lack of theory of mind or lack of central coherence and then using these to decide the presence of the behavioural criteria’ [48].

The RCSLT guideline [41] differed from most by suggesting consideration of theories relating to the triad of social impairments, such as executive functioning deficits, motivation, memory and central coherence, as well as social interaction and communication. However, some (e.g. [40]) suggested cognitive or neuropsychological testing whilst SIGN guidelines stated that such assessments are ‘useful for individual profiling but are not diagnostic instruments’ [10]. This anomaly may reflect the specialist role of SLTs in the diagnostic process.

Overall, we would concur with a reflection in one guideline, which noted how the HCP may be faced with ‘possible uncertainty as to where to go next in their investigation framework as this could be potentially enormous’ [53].

#### Diagnostic tools

Recommendations about the use of diagnostic tools were mixed. One third of the guidelines (*n* = 7) did not specify any particular tool for diagnostic assessment. Other CPGs tended to suggest the consideration of a range of tools without specifically recommending any particular instrument(s), although regular references were made to ADOS (*n* = 13), ADI-R (*n* = 11), DISCO (*n* = 9) and 3di (*n* = 6). The NICE guideline for children and young people emphasised use of DSM/ICD criteria rather than tools; the NICE guideline for adults did the opposite [9, 39]. Overall, findings concurred with Penner et al. in that guidelines varied substantially in their recommendations for use of diagnostic tools [29].

#### Diagnosis and formulation

There were differences in the way guidelines described the relationship between, or referred to, diagnosis, assessment, profiling, needs assessment and wider formulation. All guidelines encompassed the concept of a wider (needs related) assessment but few explicitly separated out these processes or discussed how this related to a diagnostic assessment. One exception to this was the Regional Autistic Spectrum Disorder Network (RASDN) children’s guideline, which separated the diagnostic from the formulation process, describing the latter as including examination of the person’s wider environment:

‘The outcome of the formulation should be to understand an individual in a more global holistic way rather than merely in terms of signs and symptoms, as in the case of diagnosis’ [44].

The RCPsych guideline suggested that diagnosis is only one component of the wider multidisciplinary exercise [11]. Some guidelines did not mention formulation but suggested a profile of strengths, abilities and weaknesses should be carried out alongside a diagnostic assessment (e.g. [10, 39]). Adult guidelines from RASDN separated out a diagnostic assessment from a full needs assessment [54]; NICE guidelines for adults considered comprehensive assessment to include diagnostic, needs and risk assessment [9]; whilst the full children’s guidelines similarly brought together the diagnostic and needs elements under ‘autism diagnostic assessment’, explaining that:

‘..the label of autism does not constitute a complete diagnostic assessment and a profile of the child or young person’s strengths and weaknesses is also essential. This requires a multidisciplinary team which has the skills to undertake the assessments necessary for profiling’ [55].

Operationally, therefore, there were contradictions between guidelines about what constitutes the diagnostic process, how it should be structured and which diagnostic tools should be used.

### Interactional factors

Interactional factors related to how the dialogue between HCPs and between HCPs and families impacts on the assessment process. These include how consensus is reached, how disagreement is resolved and how the views of the person and family are integrated into the decision-making process.

#### Multidisciplinary assessment versus single practitioner assessment

Where specified, all guidelines advocated for diagnosis to take place within a multidisciplinary setting with various guidelines suggesting this was ‘necessary’ [44], the ‘optimum approach’ [10] or ‘ideal’ [43] (See Table 4). Some suggested (*n* = 4) that an appropriately trained and experienced single professional is sufficient to diagnose in particular cases, but to be alert for indications for a more specialist assessment [11] and with access to multidisciplinary support if required [48].

Despite this almost universal recommendation, the extended version of NICE children’s guidelines (and cited by SIGN [10] and Carpenter [48]) questioned the evidence base for multidisciplinary assessment reporting a study [56] that showed moderate agreement between an individual HCP and an MDT in making a diagnosis, but stating that it was a low quality study [55]. These guidelines also suggested in practice that a diagnosis can be made by a single experienced HCP but that a comprehensive profile of the patient requires a multidisciplinary approach [55]. SIGN guidelines also cited research [57] which demonstrates that parents value a multidisciplinary assessment [10].

None of the guidelines in this review dealt with how HCPs come to a consensus within a multi-disciplinary context, although Northern Ireland guidelines recommended that training should include the promotion of collaborative and innovative working [44] and that clinicians must understand the profession specific roles and responsibilities of the overall team [44, 54].

Therefore, most guidelines referred to MDTs as best practice, but lacked recommendations about how roles within MDTs are negotiated, how disagreement is resolved (other than second opinion outside the team); or how teams should work together, a factor that is acknowledged by NICE adults guideline [9].

#### Interaction with the person and their family

Many guidelines (*n* = 9) outlined the importance of keeping the person/family informed and involved throughout the process or recommended a person-centred approach. Some described the relationship with the person coming for diagnosis and their family as a partnership (e.g. NICE adult guideline [9]) or as person-centred (e.g. RASDN adult guideline [54]). Some guidelines (*n* = 6) acknowledged that the person or family may disagree with or be reluctant to accept a diagnosis or, alternatively desire one [46] and be determined on a particular outcome, which can lead to misleading results [11]. Carpenter asserted that some people may begin to see diagnosis as a desirable outcome and pre-prepare answers based on structured interviews published on the internet [48]. The potential for disagreement or desire for diagnosis, therefore, may impact on the interaction with the person or their family. So, although the relationship with the patient/family is considered within CPGs, there is little guidance as to how HCPs might deal with patient/family desire or disagreement.

### Contextual factors

There were factors related to the way in which HCPs interpret symptoms in different settings, how diagnostic thresholds are judged against criteria and included considerations around the impact and consequences of a diagnosis.

#### Interpreting needs

All national guidelines (*n* = 5) outlined the requirement to consider the needs, preferences and values of the individual and their family and/or support them to communicate their needs and concerns. Most guidelines (*n* = 17) described elements of diagnosis that relate to either family environment, family needs and concerns, circumstances, relationships, functioning, experiences in different settings, contextual information or level of support needs. Many guidelines reflected the need to consider assessment of support required. Enquiries should be made about how symptoms impact on function within the family, at home, school or work [9, 39, 47, 54, 58]. Overall, therefore, there was a focus not only on the assessment of symptoms, and the way in which these affect the daily life of the person and their family, but the wider environmental and social context of the person and the way in which they are supported, or not, by that context.

#### Masking and social context

Some guidelines (*n* = 6) reported the difficulties of diagnosing autism when compensation strategies may ‘mask’ difficulties in some contexts, particularly as an adult [33], and in girls [50] where autism may go unrecognised. Some suggested that individuals may come forward for diagnosis when their circumstances change and/or stressors increase (e.g. [10, 45, 54]). Some guidelines (*n* = 5) noted that cultural differences will exist in norms for social interaction or that cultural variations can deliver misleading signs and symptoms. DSM-5 suggested that the boundaries between normality and pathology differ between cultures and the level at which experience may become problematic may differ [33].

SIGN suggested that those with autism may not have met ‘normal’ adult milestones in work, relationships or independence and contained extensive information on how females can present with a different symptom profile [10]. Others warned that behaviours might be the result of disruptive home experiences, carer illness [39] or complex psychosocial or child protection backgrounds [53].

Despite research showing links between diagnostic rates and SES, there was very little mention of the impact of SES in CPGs. DSM-5 stated that cultural and socioeconomic factors may affect age at recognition or diagnosis [33] but generally guidelines failed to consider how this might be considered in practice, other than to be aware that ‘cultural variations can deliver misleading signs and symptoms’ [45] or that autism is ‘not restricted to particular ethnic or economic backgrounds’ [40]. RCSLT guidelines considered assessment of bilingual individuals [41] and some suggested that ethnicity may delay engagement in the diagnostic process [11] or may increase difficulty in accessing services [54].

Overall, guidelines suggested it was the responsibility of the HCP to make a judgement about which behaviours appear to be ‘normal’ in complex social and family circumstances, as well as against norms for behaviour.

#### Diagnostic uncertainty, thresholds and the role of clinical judgement

Overall the general focus of guidelines was to outline a framework to find the best way to decide whether autism is present or not around a threshold of symptom severity. However, many guidelines problematized this, for example, one guideline discussed how definitions of autism have changed with DMS-5 [11] and others suggested that social factors, such as an upbringing characterized by lack of boundaries [45] or symptoms amplified by distress [11] may cause diagnostic difficulties.

All national guidelines considered uncertainties around diagnosis, particularly with very young children or those with co-existing disorders [39]; when there may be disagreement within the diagnosing team or between the team and the patient or family, or when there is a lack of local expertise [9]. Many warned of diagnostic difficulties, or ‘obscuring’ [11, 53] that can take place if there is an intellectual disability or other complex coexisting condition and several considered the difficulties of overlapping diagnostic criteria [33, 50, 51, 53]. Further uncertainty was outlined when individuals may not reach the diagnostic threshold [39] or when children with autism score below the cut-off as determined by the diagnostic instrument [43].

Despite this uncertainty, CPGs generally proposed a systematic approach to diagnosis and, in some cases, asserted that progress has been achieved in establishing consensus around a behavioural definition and established systematic clinical assessments (e.g. [50]) even whilst recognizing that the ‘boundaries between disorders are more porous than originally perceived’ [33].

Eight guidelines stressed the key role of clinical judgement in the diagnostic process. DSM-5 outlined that the use of diagnostic criteria should be informed by clinical judgement [33] and ICD-10 suggested that guidelines should be used flexibly in clinical work [32]. The full version of NICE children’s guideline recommended: ‘Use information from all sources, together with clinical judgement, to diagnose autism based on ICD-10 or DSM-IV criteria’ [55]. One guideline suggested that clinicians may depend on the ‘feel’ of the interaction with the patient for diagnosis [48]. The RCPsych guideline stated that:’…much will depend on the extent of the clinician’s experience, their rigour in applying standard criteria and their ability to recognise alternative diagnoses’ [11]. Uncertainty, clinical judgement and clinician experience, therefore, were all identified as important factors in the diagnostic process.

#### Pragmatic outcomes and diagnostic value

Most guidelines (*n* = 17) discussed the need for HCPs to have knowledge of local support and resources available to deliver appropriate advice when required. The value of the diagnosis was generally described as a way to provide appropriate support, intervention and resources. NICE guidelines for children and young people clarified this:

‘Diagnosis and the assessment of needs …can open doors to support and services…all of these can improve the lives of the child or young person and their family’ [39].

However, NICE guidelines for adults acknowledged that adults who are diagnosed may receive no support due to lack of services [9] and Pilling et al. stated that whilst care for children and young people is generally well coordinated, this is not always the case for adult services [58].

Although some guidelines acknowledged that people may not want a diagnosis and the label it brings with it (e.g. [44, 54]) or that it can be stigmatising or damaging to career plans [11], generally guidelines described the benefits of a diagnosis primarily as relating to improved quality of life, creating an opportunity to have needs met, greater understanding and reassurance about one’s own situation and access to interventions and services. Some guidelines considered that diagnosis can provide relief, understanding or an opportunity to move on with increased support [11, 39, 45].

Many guidelines stressed the importance of early diagnosis as this enables early intervention which leads to improved health outcomes (e.g. [41, 44, 47, 50]). However, the BMJ guideline asserted that the, ‘…efficacy of early intervention varies from child to child’ [43], and that ‘consideration of the direct financial costs, indirect costs… and the impact on relationships within the family… must be balanced against likely and possible improvements in outcome for the person with ASD’ [43], bringing uncertainty into the benefits of diagnosis. Furthermore, O’Hare asserted that it is difficult to prove that earlier intervention is more effective [53].

Overall, guidelines reflected a concern about the potential impact or benefits on the child or adult receiving a diagnosis and considered positive factors such as access to support and intervention, increased understanding or relief; as well as potential negative impacts such as stigma. Carpenter, however, questioned the relationship between need and diagnosis, by asking whether diagnosis is influenced by what intervention the person needs or ‘…explicitly determined by the person’s need to have the label to access a service… rather than their fitting strict diagnostic criteria?’ [48].

To conclude, whilst CPGs appeared to frame a methodical and clinical diagnostic process, they also rehearsed a number of subjective dilemmas that HCPs have to negotiate along the way. Some CPGs themselves drew attention to social issues that muddle the process: the difficulties of establishing a clear threshold in a condition where symptoms are impacted by the stressors of environment [11]; the problem of relying on mechanistic assessments or algorithms [11, 48]; the crucial role of clinical judgement [54]; the possibility of diagnostic uncertainty through disagreement, lack of local expertise or when a complex coexisting condition is present [9]; the complexity caused by interaction with co-occurring conditions; masking of autism by comorbid conditions in secondary care [58]; the impact of good (or poor) social support and coping strategies on how symptoms present [33], to name a few.

## Discussion

We found that CPGs varied in how they described the diagnostic process in relation to use of diagnostic tools, key elements and structure of the diagnostic process (for example how diagnosis related to wider needs assessment) and how autism was classified, defined either by current versions of DSM or ICD. In addition, whilst some recommendations were clear and universal, for example, recommendations for multidisciplinary working, there was little guidance as to how this should work in practice.

In addition, we found that uncertainty was central to many diagnostic decisions, placing a great emphasis on clinical judgement. This uncertainty included questions around the benefits of early intervention, the shifting nature of the diagnostic threshold, the difficulties of interpreting needs in different social contexts, the problems of interpreting ‘masking’ or coping strategies, the differences in presentation across age and breadth of symptoms, the inter-relationship with co-conditions and sharing of symptoms, the impact of stressors on symptoms as well as interpretation of symptoms and needs in different cultural contexts.

Overall, therefore, our narrative review found that although individual guidelines appeared to present a coherent and systematic assessment process, they varied enough in their recommendations to make the choices available to healthcare professionals particularly complex and confusing; and presented a context of uncertainty which appeared to be central to the diagnosis of autism. We argue that clinical guidelines for autism diagnosis illuminate the process of diagnosis as social rather than straightforwardly clinical, and that judgement is required to consider a number of sometimes contradictory and complex social factors.

### Social factors in CPGs

Organising the narrative review findings in relation to operational, interactional and contextual factors enabled consideration of the influence of social factors throughout the diagnostic process.

In the wide range of inter-related assessment processes that HCPs negotiate in order to make the diagnostic decision, the factors considered appear to be both social and medical. Social factors include: how the category of ‘autism’ is defined and boundaried; operational and interactional factors present in the process of diagnosis; to the consequences of diagnosis including how diagnosis is valued (see Fig. 3). Each of these factors had a place in clinical guidelines to a greater or lesser extent but in many cases they were not operationalized to enable a clear and transparent framework. For example, although there were many references to individuals masking symptoms, family ‘scaffolding’ of social impairment and coping strategies, there was little guidance about how HCPs can judge the impact of these on need, behavioural symptoms or functioning.

CPGs, therefore, tended to mask (whilst paradoxically acknowledging) the existence of social factors in the diagnostic process. A more explicit acknowledgement of social factors and how to manage them might problematize the nature of autism diagnosis altogether: if all these factors have a place in diagnosis, how do they relate to clinical factors and what does it mean for descriptions of symptoms? Whilst it is not our intention to undermine the utility of diagnostic categories in relation to access to resources or support, there appears to be a need for balance in CPGs between a clinical approach which both recognises and acknowledges the uncertainty of the diagnostic threshold; and a pragmatic or functional approach which responds to individual and wider needs and takes account of social factors.

### Diagnostic tools and process

Clinical guidelines for autism varied in aspects of their key recommendations in operational factors. Ambiguities around which tools to use, the key elements in the diagnostic process and the relationship between diagnosis, assessment and formulation suggest that local practice may be shaped by other factors, such as available resources, experience and professional roles. Which tools are used, whether different elements of the process are considered together, sequentially or inconsistently, and the specific aims of each part of the assessment process may have an impact on diagnostic outcomes. A clearer framework would help HCPs to consider which elements of the process are relevant and when.

### MDT working and views of the family

Guidance about how HCPs can reach a consensus with others in a multidisciplinary context or deal with patient/family disagreement or desire was lacking, leaving interactional factors as key to the process but largely unexplained. Whilst it might not appear to be in the remit of CPGs to make specific recommendations about how teams are organised and configured, particularly across different health systems, we argue that team functioning as a key shaping factor in diagnosis requires more attention in CPGs, to ensure clarity of roles and transparency for those coming for diagnosis. Similarly, as acknowledged by some CPGs, desire of the patient/family can influence the diagnostic process, therefore CPGs should offer guidance about how that might be managed.

### Diagnostic uncertainty and judgement

Uncertainty about diagnostic thresholds and differences in diagnostic criteria make clinical judgement key to the diagnostic process and yet how this comes about was not clearly defined. The extent to which diagnosis should be based on underlying symptoms versus contextual factors such as wider needs or circumstances of the individual was unclear. In addition, how HCPs consider the consequences of the diagnosis for the patient and their family was unclear, although there was a strong link described between diagnosis and access to support.

Ambiguities in CPGs suggest that guidelines have limitations in how far they are able to promote consistency across practice especially given the lack of a biomarker for autism, the reliance on observed behaviour and family narratives for diagnosis, and the differences across health systems. However, adults, children and families coming for diagnosis might expect a consistent process of assessment in keeping with a framework outlined in CPGs, as CPGs become a fixed reference point both for HCPs and the lay public. There is, therefore, a tension between potential expectations of those coming for diagnosis that there should be a uniform process; and the flexibility HCPs require to respond to individual need.

Given the social nature of diagnosis as argued in this article, biomarker use in clinical practice, if and when it is successfully developed, is likely to remain only one aspect of an interactive diagnostic process, and therefore may not necessarily alleviate some of the difficulties and complexities of diagnosis that we describe. However, as biomarker research develops, it is likely that it will produce important evidence to be considered in the development of future CPGs.

### Building on previous work

Whilst our narrative review differed in purpose to the systematic review undertaken by Penner et al. [29], there were some similar findings across the two studies. We found, as did the authors of this previous review, that guidelines were inconsistent in their recommendations around diagnostic assessment. For example, whilst guidelines generally recommended MDT assessment, some suggested that a single experienced clinician could diagnose [11, 39, 48] and there was little cited evidence for the efficacy of MDT assessment. In addition, CPGs did not provide guidance as to how waiting times (where specified) would be achieved and we would add that they provided little operational guidance as to how MDT decision-making should operate to be most effective. We found, as did Penner et al., that guidelines varied substantially in recommended tools and personnel; and that none of the professional guidelines provided target waiting times for assessment (See Table 4). Whilst we did not assess guidelines for quality, we agree that there are multiple guidelines that HCPs might access, and that they vary in their level of detail and their recommendations.

We built on Penner et al.’s findings in a number of ways. Our review of the range of assessment processes that HCPs involved in autism diagnosis may undertake (See Table 4) suggested a wide range of choices in assessment processes. We also found that using different classification criteria (ICD-10 and DSM-5) further increases complexity in CPGs. Finally, we found that consideration of factors such as interaction with the patient and family, how needs might be defined and assessed, and issues of masking, social context, uncertainty and clinical judgement highlighted the way in which social processes and factors might impact on diagnostic decision-making. We also found that, despite the CPGs in our study operating within comparable health systems across the UK, CPGs did not make consistent recommendations around how diagnosis might release post-diagnostic resources, and what that means for the process of diagnosis itself.

Overall we agree with Penner et al.’s findings that CPGs should incorporate flexibility to ensure that individual needs are met. Additionally, we suggest that guidelines should acknowledge more explicitly the social framing of diagnosis and support clinicians with a framework which enables them to act pragmatically in the best interests of the patient. We would argue that inconsistencies and lack of operational guidance around social factors in CPGs suggests that local factors such as access to resources and HCP expertise are likely to shape diagnosis more than is explicitly outlined in CPGs.

Unlike Penner et al., we do not think that a formal approach to decision-making such as the Delphi method would help HCPs in the assessment process; rather it might simply add another layer of complexity to a process which is already challenging. Our experience is that HCPs already struggle to find time to meet together in the context of an ever-increasing workload; an extra administrative burden may make this even more difficult.

Finally, unlike Penner et al., we included in our review CPGs for adult diagnosis and children over 6 years old, which enabled us to consider factors common across age groups. Whilst we did not specifically look for differences between children’s and adult’s CPGs we are aware that the different pathways for children’s and adult’s assessment [3, 59], may well impact on an individual’s ability to access diagnostic services, the process of assessment itself as well as potential support post-diagnosis. We would consider these differences as social organisational factors that may impact the assessment process and merit further consideration in the development of future CPGs.

Guidelines, therefore, appear to offer a relatively linear and straightforward pathway towards a diagnostic decision in their presentation, with DSM-5 asserting that criteria facilitate an objective assessment of symptom presentations in a variety of settings [20]. However, comparing individual guidelines suggests inconsistencies in this framework and close analysis reveals a more fluid process, disrupting the apparent clinical purity of diagnosis [37].

## Conclusion

Overall, there was a bewildering range of options for HCPs in the assessment process, and a number of different emphases in guidelines which might lead a clinical team one way or another. Navigating this framework in practice is, therefore, likely to be less systematic than the guidelines might suggest, allowing for, as it must, social and contextual influences. In reality, the clinical pathway for autism diagnosis differs across health systems and trusts across the UK [3] leading to the potential for a great deal of variation in diagnostic decision-making.

### Strengths and limitations

Although there has been a recent systematic review of clinical guidelines [29], we consider our narrative approach to be helpful to understand the complex and sometimes contradictory nature of the diagnostic process. Methodologically, we undertook a systematic search and included a transparent but pragmatic selection of documents. This is, to our knowledge, the first review which strives to consider where social factors are considered in clinical guidelines for autism diagnosis. One limit was that as it was a review of current guidelines, changes through time were not exposed. Our review was limited to the UK context because health care settings vary widely in international contexts. In addition, we only examined the content of guidelines rather than how they are used. Whilst CPGs are intended to assist clinical decision-making by improving effectiveness and decreasing variations in clinical practice [60], one review of guidelines for psychiatric diagnoses suggested that CPGs are not implemented enough in clinical practice due to either lack of agreement or ambiguity between guidelines [61]. It is likely that there is wide variation in how CPGs are used in practice in autism diagnosis and we plan further studies to consider this.

### Implications and recommendations for future research

Social factors were not only explicit in guidelines, but were central to them. However, an observer might be forgiven for assuming these are subsidiary factors in diagnosis, with the more ‘medical’ ‘symptom checklist’ at its core. HCPs are expected, as outlined in DSM-5, to integrate the social, psychological and biological in case formulation, however, greater clarity about how this should operate would be helpful. Our findings suggest that more detail about how clinical judgement should consider social factors in diagnosis would provide a more transparent guideline for HCPs.

We would not recommend greater rigidity within CPGs when evidence for best diagnostic practice is inconsistent (e.g. use of diagnostic tools), and which may restrict HCPs in making decisions that are in the best interest of the person coming for diagnosis. Rather we recommend a more explicit acknowledgement of social factors in CPGs with advice about how they should be managed and operationalised to enable more consistency of practice and transparency for those coming for diagnosis.

Specifically, greater clarification is required related to the sequence and timing of the diagnostic, assessment and formulation processes. The recognition and assessment of needs is both part of the assessment process and inextricably linked to the consequences of diagnosis; guidelines might attempt to consider how these might be reconciled. A greater acknowledgement of the active role of the patient, client or patient’s family in the diagnostic process would help to place potentially competing narratives into context. It would be useful to consider whether guidelines are culturally specific to health services and setting and we would recommend that further narrative reviews should be conducted to examine CPGs in other countries. In addition, greater clarity is required around how multidisciplinary interaction might operate to support consensus decision-making. Further research creating an evidence base on best practice for multidisciplinary decision-making and the use of different diagnostic tools in practice is required, taking into account the complexity of social factors in diagnosis.

## Additional file

Additional file 1: Data Extraction Framework. (PDF 298 kb)

## Abbreviations

3di: The Developmental, Dimensional and Diagnostic Interview
ADI-R: Autism Diagnostic Interview Revised
ADOS: Autism Diagnostic Observation Schedule
BMJ: British Medical Journal
BPS: The British Psychological Society
CPG: Clinical Practice Guideline
DISCO: The Diagnostic Interview for Social and Communication Disorders
DSM-5: Diagnostic and Statistical Manual of Mental Disorders (Fifth Edition)
HCP: Healthcare Professional
HMIC: The Healthcare Management Information Consortium
ICD-10: International Classification of Mental and Behavioural Disorders (Tenth edition) ICIDH-2 International Classification of Functioning, Disability and Health
MDT: Multidisciplinary Team
NICE: The National Institute for Health and Care Excellence
RASDN: The Regional Autistic Spectrum Disorder Network
RCPsych: Royal College of Psychiatrists
RCSLT: Royal College of Speech and Language Therapists
SIGN: The Scottish Intercollegiate Guidelines Network
SLT: Speech and Language Therapist

## References

[1] Kobeissy F, Alawieh A, Mondello S, Boustany RM, Gold MS. Biomarkers in psychiatry: how close are we? Frontiers in Psychiatry. 2013;3 10.3389/fpsyt.2012.00114 PMC3539768 23316174

[2] Klin A, Lang J, Cichetti D, Volkmar F (2000). Brief Report: Interrater reliability of clinical diagnosis and DSM-IV criteria for autistic disorder: results of the DSM-IV Autism field trial. J Autism Dev Disord.

[3] Vllasaliu L, Jensen K, Hoss S, Landenberger M, Menze M, Schütz M (2011). Diagnostic instruments for autism spectrum disorder (ASD) (protocol).

[4] Huerta M, Lord C (2012). Diagnostic evaluation of autism spectrum disorders. Pediatr Clin N Am.

[5] Gillberg C. The ESSENCE in child psychiatry: Early Symptomatic Syndromes Eliciting Neurodevelopmental Clinical Examinations. Res Dev Disabil. 31:1543–51. 10.1016/j.ridd.2010.06.002. 10.1016/j.ridd.2010.06.002 20634041

[6] Falkmer T, Anderson K, Falkmer M, Horlin C (2013). Diagnostic procedures in autism spectrum disorders: a systematic literature review. Eur Child Adolesc Psychiatry.

[7] Woolfenden S, Sarkozy V, Ridley G, Williams K (2011). A systematic review of the diagnostic stability of Autism Spectrum disorder. Res Autism Spectr Disord.

[8] Field M, Lohr K (1992). Guidelines for clinical practice: from development to use. Washington DC: Institute of Medicine.

[9] NICE (2012). Autism Spectrum disorder in adults: diagnosis and management.

[10] Scottish Intercollegiate Guidelines Network. SIGN 145: assessment, diagnosis and interventions for autism spectrum disorders: a national clinical guideline. 2016. http://www.sign.ac.uk/assets/sign145.pdf. Accessed 27 Mar 2017.

[11] Royal College of Psychiatrists. Good practice in the management of autism (including Asperger syndrome) in adults: College Report CR191. 2014. http://www.rcpsych.ac.uk/files/pdfversion/CR191.pdf. Accessed 7 Jun 2017.

[12] Fuat A, Hungin APS, Murphy JJ (2003). Barriers to accurate diagnosis and effective management of heart failure in primary care: qualitative study. Br Med J.

[13] Mazumdar S, Winter A, Liu KY, Bearman P (2013). Spatial clusters of autism births and diagnoses point to contextual drivers ofincreased prevalence. Soc Sci Med.

[14] Liu K-Y, King M, Bearman PS (2010). Social influence and the autism epidemic. Am J Sociol.

[15] Skellern C, Schluter P, McDowell M (2005). From complexity to category: responding to diagnostic uncertainties of autistic spectrum disorders. J Paediatr Child Health.

[16] Rogers CL, Goddard L, Hill EL, Henry LA, Crane L (2016). Experiences of diagnosing autism spectrum disorder: a survey of professionals in the United Kingdom. Autism.

[17] Russell G, Rodgers LR, Ukoumunne OC, Ford T (2014). Prevalence of parent-reported ASD and ADHD in the UK: findings from the millennium cohort study. J Autism Dev Disord.

[18] King M, Bearman P (2011). Socioeconomic status and the increased prevalence of Autism in California. Am Sociol Rev.

[19] The National Autistic Society (2014). Diverse perspectives: the challenges for families affected by autism from black.

[20] Norbury CF, Sparks A (2013). Difference or disorder? Cultural issues in understanding neurodevelopmental disorders. Dev Psychol.

[21] Russell G, Steer C, Golding J (2011). Social and demographic factors that influence the diagnosis of autistic spectrum disorders. Soc Psychol Psychiatr Epidemiol.

[22] Goldani AAS, Downs SR, Widjaja F, Lawton B, Hendren RL (2014). Biomarkers in autism. Front psychiatry.

[23] Anwar A, Abruzzo PM, Pasha S, Rajpoot K, Bolotta A, Ghezzo A, et al. Advanced glycation endproducts, dityrosine and arginine transporter dysfunction in autism - a source of biomarkers for clinical diagnosis. Mol Autism. 2018;9 10.1186/s13229-017-0183-3. 10.1186/s13229-017-0183-3 PMC5817812 29479405

[24] Carlisi CO, Norman L, Murphy CM, Christakou A, Chantiluke K, Giampietro V (2017). Disorder-specific and shared brain abnormalities during vigilance in Autism and obsessive-compulsive disorder. Biol Psychiatry Cogn Neurosci Neuroimaging.

[25] Anderson GM (2015). Autism biomarkers: challenges, pitfalls and possibilities. J Autism Dev Disord.

[26] NHS Choices (2018). New blood test for Autism a long way off.

[27] Imran N, Chaudry MR, Azeem MW, Bhatti MR, Choudhary ZI, Cheema MA (2011). A survey of Autism knowledge and attitudes among the healthcare professionals in Lahore, Pakistan. BMC Pediatr.

[28] Taylor LJ, Eapen V, Maybery MT, Midford S, Paynter J, Quarmby L (2016). Diagnostic evaluation for autism spectrum disorder: a survey of health professionals in Australia. BMJ Open.

[29] Penner M, Anagnostou E, Andoni LY, Ungar WJ. Systematic review of clinical guidance documents for autism spectrum disorder diagnostic assessment in select regions. Autism. 2017:136236131668587. 10.1177/1362361316685879. 10.1177/1362361316685879 PMC6039866 28548543

[30] Moher D, Liberati A, Tetzlaff J, Altman DG, Group TP (2009). Preferred reporting items for systematic reviews and meta-analyses: the PRISMA statement (reprinted from annals of internal medicine). Phys Ther.

[31] Liberati A, Altman DG, Tetzlaff J, Mulrow C, Gøtzsche PC, Ioannidis JPA, et al. The PRISMA statement for reporting systematic reviews and meta-analyses of studies that evaluate health care interventions: explanation and elaboration. PLoS Med. 2009;6 10.1371/journal.pmed.1000100. 10.1371/journal.pmed.1000100 PMC2707010 19621070

[32] World Health Organization (1993). The ICD-10 Classification of Mental and Behavioural Disorders: diagnostic criteria for research.

[33] APA (2013). Diagnostic and statistical manual of mental disorders.

[34] Popay J, Roberts H, Sowden A, Petticrew M, Arai L, Rodgers M, et al. Guidance on the Conduct of Narrative Synthesis in Systematic Reviews: A Product from the ESRC Methods Programme. 2006. https://www.researchgate.net/profile/Lisa_Arai/publication/242311393_Guidance_on_the_conduct_of_narrative_synthesis_in_systematic_reviews_a_comparison_of_guidance-led_narrative_synthesis_versus_meta-analysis/links/5532159f0cf2f2a588ad67fd.pdf. Accessed 20 Jun 2017.

[35] Ferrari R (2015). Writing narrative style literature reviews. Med Writ.

[36] Jutel A, Nettleton S (2011). Towards a sociology of diagnosis: reflections and opportunities. Soc Sci Med.

[37] Latimer J. The gene, the clinic and the family: diagnosing dysmorphology, reviving medical dominance. Oxfordshire: Routledge; 2013.

[38] Department of Health (2010). “Fulfilling and rewarding lives”: The strategy for adults with autism in England.

[39] NICE. Autism spectrum disorder in under 19s: recognition, referral and diagnosis. 2011. https://www.nice.org.uk/guidance/cg128/resources/autism-spectrum-disorder-in-under-19s-recognition-referral-and-diagnosis-pdf-35109456621253. Accessed 2 Nov 2016.

[40] British Psychological Society. Autistic Spectrum Disorders: Guidance for Psychologists. Br Psychol Soc. 2016; www.bps.org.uk. Accessed 27 Mar 2017 Available at https://blogs.exeter.ac.uk/exploringdiagnosis/.

[41] RCSLT. Royal College of Speech & Language Therapists Clinical Guidelines. Oxon: Speechmark; 2005. p. 63–71.

[42] World Health Organisation (2001). International classification of functioning, Disability and health.

[43] Parr J, Woodbury-Smith M. Autism spectrum disorder. BMJ Best Pract. 2017:59–83. https://bestpractice.bmj.com/topics/en-gb/379.

[44] RASDN. Six Steps of Autism Care. 2011. http://www.belfasttrust.hscni.net/pdf/Six_Steps_of_Autism_Care_Pathway_Report.pdf. Accessed 25 Mar 2018.

[45] Howlett D, Richman S (2011). Recognising, referring and diagnosing autism. Every Child J.

[46] Reynolds KE (2011). Autism spectrum disorders in childhood: a clinical update. Community Pr.

[47] Blenner S, Reddy A, Augustyn M. Diagnosis and management of autism in childhood. BMJ. 2011; 10.1136/bmj.d6238. 10.1136/bmj.d6238 22021467

[48] Carpenter P (2012). Diagnosis and assessment in autism spectrum disorders. Adv Ment Heal Intellect Disabil.

[49] Garland J, O’Rourke L, Robertson D (2013). Autism spectrum disorder in adults: clinical features and the role of the psychiatrist. Adv Psychiatr Treat.

[50] Lai M-C, Lombardo MV, Baron-Cohen S (2014). Autism. Lancet.

[51] Levy SE, Mandell DS, Schultz RT (2009). Autism. Lancet.

[52] Wilson CE, Roberts G, Gillan N, Ohlsen C, Robertson D, Zinkstok J (2014). The NICE guideline on recognition, referral, diagnosis and management of adults on the autism spectrum. Adv Ment Heal Intellect Disabil..

[53] O’Hare A (2009). Autism spectrum disorder: diagnosis and management. Arch Dis Child - Educ Pract.

[54] Regional Autistic Spectrum Disorder Network. Autism Adult Care Pathway. 2013. http://www.hscbusiness.hscni.net/pdf/Adult_Care_PAthway_Reviewed_August_2013.pdf. Accessed 21 Jun 2017.

[55] National Collaborating Centre for Women’s and Children’s Health. Autism: recognition, referral and diagnosis of children and young people on the autism spectrum: NICE Clinical Guideline. 2011. https://www.ncbi.nlm.nih.gov/pubmed/22624178. Accessed 17 Feb 2017.

[56] Mahoney WJ, Szatmari P, MacLean JE, Bryson SE, Bartolucci G, Walter SD, Jones MB, Zwaigenbaum L (1998). Reliability and accuracy of differentiating pervasive developmental disorder subtypes. J Am Acad Child Adolesc Psychiatry.

[57] Moore V, Titcomb J, Johnson C, Cronk E, Baker S, Thysson L (1998). Developing an autism assessment service II: analysis of the first 81 cases seen. Child Psychol Psychiatr Rev.

[58] Pilling S, Baron-Cohen S, Megnin-Viggars O, Lee R, Taylor C (2012). Recognition, referral, diagnosis, and management of adults with autism: summary of NICE guidance. BMJ.

[59] Baird G, Douglas HR, Murphy MS. Recognising and diagnosing autism in children and young people: summary of NICE guidance. BMJ. 2011;343:–d6360. 10.1136/bmj.d6360 22021468

[60] Kredo T, Bernhardsson S, Machingaidze S, Young T, Louw Q, Ochodo E (2016). Guide to clinical practice guidelines: the current state of play. Int J Qual Heal Care.

[61] Saddichha S, Chaturvedi SK (2014). Clinical practice guidelines in psychiatry: more confusion than clarity? A critical review and recommendation of a unified guideline. ISRN Psychiatry.

[62] National Collaborating Centre for Mental Health. Autism: the NICE guideline on recognition, referral, diagnosis and management of adults on the autism spectrum. In: The British Psychological Society and the Royal College of Psychiatrists; 2012. https://www.nice.org.uk/guidance/cg142/evidence/full-guideline-186587677.

