# Supplementary material for: Clinical practice guidelines for diagnosis of autism spectrum disorder in adults and children in the UK: a narrative review
Source: BMC Psychiatry. 2018 Jul 13;18:222. doi: 10.1186/s12888-018-1800-1 (PMC6044100; doi:10.1186/s12888-018-1800-1)
Supplement: Supplementary file 1 — Data Extraction Framework. (PDF 298 kb) [file 12888_2018_1800_MOESM1_ESM.pdf]

Additional File 1: Data Extraction Framework

SECTION A: document characteristics

A1 Name of document

A2 Author

A3 Year of publication

A4 Journal title/publisher

A5 Geographical remit

A6 Type of document (practice parameter, clinical guideline etc.)

A7 Status of document (statutory/guidance only etc.)

A8 Who is the guidelines target audience?

A9 What age range (of patients) does the document encompass?

A10 Range of diagnoses covered by document

A11 Legal Framework on which guidance is based?

SECTION B: Pre-diagnosis and context for diagnosis

DO THE GUIDELINES OFFER RECOMMENDATIONS RELATED TO THE FOLLOWING?

B1 Who can refer to autism team?

B2 Which professionals can provide a diagnosis?

B3 What training or experience of clinicians is required to diagnose?

B6 Is there a stated or recommended age at which symptoms can be assessed/recognised?

B4 Is there a targeted waiting time from referral to diagnosis?

B5 Is there guidance about how to achieve any targeted waiting times?

B6 Is there additional guidance about how to deal with numbers of patients waiting and throughput?

SECTION C: assessment process

DO THE GUIDELINES OFFER RECOMMENDATIONS RELATED TO THE FOLLOWING?

C1 What should be included in a comprehensive assessment?

C2 What should a diagnostic pathway should look like?

C3 Which diagnostic criteria should be referred to (e.g. ICD/DSM-V)

C4 Which diagnostic tools (or types of tools) must be used?

C5 Whether multiple professional assessments are necessary or recommended? If so which professionals?

C6 What the role of MDT should be?

C7 How to manage the process if person has differential diagnosis/coexisting conditions?

C8 How to manage the process if person has learning disability?

C9 What to be aware of if there are culturally specific factors?

C10 Whether advice or guidance from charities or political movements is included?

C11 Whether local intervention, support or treatment resources are taken into account during decision?

C12 Whether pragmatic decisions are made related to likely outcomes for the child/patient?

C13 How to deal with if the diagnosis is **uncertain?**

C14 How to deal with if the diagnosis is **disputed?** (if patient or other clinicians disagree)

C15 How to involve the patient/family or carer in the diagnostic process or decision?

Section D: Post-diagnosis

DO THE GUIDELINES OFFER RECOMMENDATIONS RELATED TO THE FOLLOWING?

D1 When and how to discharge?

D2 How the decision should be explained to the family/patient?

D3 Recommended next steps for patient including treatment, further testing or signposting??

D4 How to support patient to deal with the potential impact of diagnosis?

D5 How do the guidelines explain the value or usefulness of a diagnosis to the patient?
